# Efficacy and tolerability of oxycodone versus fentanyl for intravenous patient-controlled analgesia after gastrointestinal laparotomy: A prospective, randomized, double-blind study

**DOI:** 10.1097/MD.0000000000004943

**Published:** 2016-09-30

**Authors:** Zhen Ding, Kaiguo Wang, Baosheng Wang, Naibao Zhou, Hao Li, Bo Yan

**Affiliations:** a School of Medicine and Life Sciences, University of Jinan-Shandong Academy of Medical Sciences; b Department of Anesthesiology, Shandong Cancer Hospital affiliated to Shandong University, Shandong Academy of Medical Sciences, Jinan, China.

**Keywords:** fentanyl, gastric laparotomy, oxycodone, patient-controlled analgesia, postoperative pain

## Abstract

**Background:**

It has been suggested that oxycodone is effective in relieving acute postoperative pain. The aim of this study was to investigate the efficacy and tolerability of oxycodone (O) versus fentanyl (F), and the adequate potency ratio of oxycodone and fentanyl in patients with intravenous patient-controlled analgesia after gastric laparotomy.

**Methods:**

In this double-blinded, randomized, controlled study, 60 patients undergoing elective gastric laparotomy were allocated to receive either oxycodone or fentanyl for postoperative intravenous patient-controlled analgesia (potency ratio 60:1). The patients received ketorolac 60 mg before the end of anesthesia and then continued with patient-controlled analgesia for 48 hours postsurgery. Pain severity, side effects and respiration rate were recorded 30 minutes, 3, 6, 12, 24, and 48 hours after the surgery. Cumulative opioid requirements and patient satisfaction were also measured.

**Results:**

The median consumption more than 48 hours after operation of oxycodone was 50 mg (range: 40.0–62.4 mg) and fentanyl was 0.8 mg (range: 0.6–1.1 mg), and the percentage of patients requiring rescue medication was not statistically significant. Numeric rating scores at rest and upon movement were significantly lower in group O than in F (*P* < 0.05). Whereas the incidences of adverse events were similar between the groups (33.3% vs 27.6%, *P* = 0.64), a significant higher sedation scores were found in patients given fentanyl at 30 minutes after the surgery (*P* = 0.04).

**Conclusion:**

Oxycodone was comparable to fentanyl in the relief of postoperative pain following gastric laparotomy. Oxycodone not only provides better postoperative pain relief and less sedation, but also there was a tendency toward more side effects with oxycodone.

## 1 Introduction

Gastric laparotomy is often accompanied by severe postoperative pain, especially the visceral components is the major challenge in the practice of clinicians dealing with patients undergoing major abdominal surgery. Increasing evidence shows that undertreated acute pain may not only increase the need for hospitalization after surgery,^[1]^ but also is a risk factor for chronic postoperative pain.^[2,3]^

Opioids are considered to be the standard analgesic for relieving moderate to severe acute postoperative pain. After surgery, however, many patients are unable to ingest oral medication and instead receive intravenous an opioid. Patient-controlled techniques allow patients to self-administer small boluses of analgesic, providing better titration and enhancing responsiveness in analgesic requirement.^[4–6]^ Patient-controlled analgesia (PCA) with intravenous an opioid is a more efficient method for achieving improved postoperative analgesia and high patient satisfaction with minimal sedation and complications,^[7]^ has become the most common regimen for pain relief after major surgery.

Fentanyl is widely used for the control of postoperative pain, but there is a trend toward the use of oxycodone. Oxycodone, a semisynthetic opioid that may be an agonist of the central and peripheral kappa- as well as mu-opioid receptors,^[8–11]^ is superior in the treatment of visceral pain.^[12,13]^ Oxycodone is highly effective and well tolerated in different types of surgical procedure and in patients with renal or hepatic failure, and age is no contraindication for oxycodone, is replacing morphine as a first-choice opioid in several countries. In china, the utility of oxycodone is increasingly frequently in patients after major abdominal surgery. To the best of our knowledge, no comprehensive data exist regarding the analgesic efficacy of intravenous oxycodone versus fentanyl in patients after gastric laparotomy.

In this prospectively, randomized, double-blind study, we compared the analgesic efficacy, adverse events, and patient satisfaction ratings of fentanyl and oxycodone in patients experiencing moderate to severe pain after gastric laparotomy. In addition, this study also evaluated the conversion dose rate of intravenous PCA (IV-PCA) oxycodone and fentanyl for postoperative analgesia on the basis of adequacy pain control.

## 2 Materials and methods

The study protocol was approved by the Institutional Review Board at Shandong Cancer Hospital affiliated to Shandong University, Shandong Academy of Medical Sciences, Jinan, China. Written informed consent was obtained from each patient or a legal representative. We collected patients in our institute from July 2015 to December 2015. Patients of age between 40 and 70 years who scheduled to undergo elective gastric laparotomy with general anesthesia were eligible if they were considered as American Society of Anesthesiologists’ (ASA) Class I to III classification and with a body mass index (BMI) 18–25 kg/m^2^. The following patients were excluded: pregnant and lactation women; allergy, sensitivity, or contraindication to study medications; history of chronic opioid usage within 8 weeks of surgery; had bleeding disorders; history of severe hepatic, renal, pulmonary, or cardiac disease; history of underlying psychological disorders or communication disability.

All patients were assigned randomly to receive either oxycodone (group O) or fentanyl (group F) by blinded researchers, according to a computer-generated simple randomization code. Group allocation was controlled and concealed by our institution's pharmacy and released only after completion of the study, thus ensuring blinding of patients, researchers, and observers. Double-blinding was achieved by labeling the PCA reservoir bags with consecutive patient numbers. For reasons of patient safety, a sealed envelope containing the treatment assignment was kept with the patient in the ward.

In the morning before the operation, the patients were instructed about the use of PCA system (CADD-Legacy 6300^®^, Smiths Medical, St. Paul, Minnesota, USA) and a numeric rating scale (0–10; 0 = no pain and 10 = worst possible pain), the purpose of the study, side effects and cautions were also explained to the patients.

Anesthesia was standardized in both groups. All patients were premeditated with 0.5 mg atropine and 1 mg phenobarbital intramuscularly approximately 30 minutes before surgery. Upon arrival in the operating room, routine hemodynamic monitoring (electrocardiogram, noninvasive blood pressure, and pulse oximetry) were used for all patients. After connecting the monitoring devices, general anesthesia was induced with propofol 1.5 to 2.0 mg/kg, fentanyl 2 to 4 μg/kg, cisatracurium 0.15 to 0.20 mg/kg, and endotracheal intubation was performed later. Anesthesia was maintained with sevoflurane 0.8% to 2.0% in 50% nitrous oxide and 30% oxygen, and a sufentanil injection of 0.2 to 0.4 μg/kg was used for analgesia as necessary. All patients were given 30 mg lansoprazole i.v. (at induction of anesthesia) and 0.125 mg of palonosetron i.v. (during operation) as prophylaxis of intraoperative regurgitation and postoperative nausea and vomiting.

At the skin suturing stage, the patients were attached to an IV-PCA pump, and 60 mg ketorolac i.v. was administered to all patients for postoperative pain relief. Neostigmine 0.2 to 0.5 mg and atropine 0.015 mg/kg were given to reverse residual neuromuscular block at the end of surgery. Tracheal extubation was performed after recovery of response to verbal commands and spontaneous respiration.

According to reviewing previous research and conducting a few pilot trials, the potency ratio of oxycodone and fentanyl were determined at 60:1. Patients in group O were administered a 100-mL mixture of oxycodone 0.7 mg/kg, 180 mg of ketorolac, and 0.125 mg of palonosetron with a normal saline solution. On the other hand, patients in group F were administered fentanyl 12 μg/kg, 180 mg of ketorolac, and 0.125 mg of palonosetron with a normal saline solution. The setting for PCA was 1 mL bolus with a 15-minutes lockout and a background continuous infusion of 1 to 2 mL/h. As soon as the patients were awake, their pain was assessed using the Numeric rating scores (NRS). If the patient reported a NRS at rest more than 3, an anesthetist monitoring and interviewing of the patients titrated PCA solution i.v. 2 mL at 5 minutes of intervals until the NRS was 3 or less. Then, the patients were encouraged to self-administer small boluses with their own PCA medications. To optimize analgesia while minimizing adverse event and hemodynamic instability, the patient-controlled setting could be further adjusted by the observer during the 48 hours of postoperative period, nurses also received extensive training regarding the studied techniques. When the patient had received the maximum number of bolus, but the NRS was higher than 3, bucinnazine 100 mg intramuscular (i.m.) were additionally administered.

Intensity of pain, level of sedation, the amount of analgesics consumed, administration of additional analgesics, side effects, and RR were evaluated and recorded at 30 minutes, 3, 6, 12, 24, and 48 hours after operation. The cumulative consumption of opioid and the overall satisfaction of patients were measured at 48 hours after the surgery.

Pain scores were evaluated with NRS in 2 categories, at rest and upon movement. Patients were asked to score their worst NRS since the previous assessment. NRS at rest was assessed with the patient lying supine and NRS upon movement was assessed with the patient were in activities including lifting limbs, sitting, rolling, or coughing.

Adverse events such as nausea, vomiting, and dizziness were investigated by incidence and severity. The severity of an adverse effects judged by observer as unrelated, possibly related, or defined related to the study drug was classified as mild (discomfort noticed, but no disruption of anticipated normal activity), moderate (discomfort sufficient to reduce or affect anticipated normal activity), or severe (inability to perform anticipated normal daily activity). When adverse events occurred, metoclopramide 10 mg i.m. were available once the patients requested. Persistent nausea, vomiting, or dizziness would warrant PCA termination with the patient then being switched to an alternate analgesic modality. PCA-related bradycardia (heart rate < 50 beat/min), respiratory depression (ventilatory frequency < 8 bpm lasting for more than 10 min) were considered as severe adverse events. If severe adverse events occurred, the use of PCA was interrupted immediately and the adverse events were treated with appropriate medication. All medications administered were recorded and this information was maintained as part of the study data.

Level of sedation was assessed with Ramsay sedation score scale (1, patient anxious and agitated or restless or both; 2, patient cooperative, oriented, and tranquil; 3, patient responds to commands only; 4, patient asleep, shows brisk response to light glabellar tap or loud auditory stimulus; 5, patient asleep, shows sluggish response to light glabellar tap or loud auditory stimulus; and 6, patient asleep, shows no response to light glabellar tap or loud auditory stimulus).

The overall satisfaction of patients with the postoperative analgesia were evaluated on a 5-point scale (1 = very unsatisfied, 2 = unsatisfied, 3 = neutral, 4 = satisfied, or 5 = very satisfied) at 48 hours postsurgery.

### 2.1 Statistical analysis

The primary outcome measure was the NRS scores (0–10). To detect a difference of 1 in NRS scores with a standard deviation of 1.2 for each group based on the previous studies, 23 subjects per group would be needed for a study with α level was 0.5 (two-tailed) and β level was 0.2 (80% power).

SPSS 17.0, Chicago, IL was used to perform statistical analyses. Patient characteristics (age, BMI, and duration of operation) and RR were analyzed using Student *t* test. Cumulative opioid consumptions, pain and sedation scores were analyzed using the Mann–Whitney *U* test. Sex, ASA classification, incidence of adverse events, use of rescue medication, and patient satisfaction were analyzed using the chi-square (*X*^2^) test or Fisher exact test. A *P* value less than 0.05 was considered to be statistically significant.

## 3 Results

A total of 60 patients were enrolled and were randomized to treatment, with 30 patients were allocated to each of the 2 groups. Four patients (6.67%) withdrew from the study: 1 patient in group O required reoperation within 24 hours of surgery for postoperative anastomotic errhysis; 2 patients in group O and F discontinued treatment because of adverse events (severe vomiting, persistent moderate upper abdominal pain); and 1 patient in group O discontinued the study due to hypotension that was judged more likely to be caused by anaemiae. Therefore, 56 patients completed the study: 27 in group O and 29 in group F. There were no statistically significant between-group differences with regard to patient clinical characteristics and intraoperative data including age, sex, BMI, ASA physical status, and duration of surgery (Table 1).

**Table 1:**
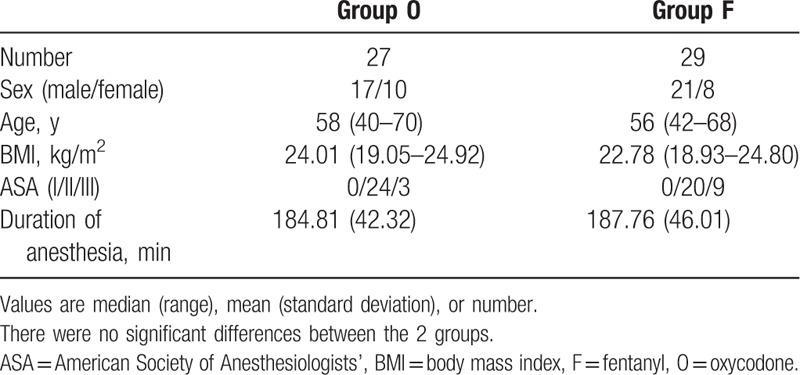
Patient clinical characteristics and intraoperative data.

The median consumption over 48 hours after operation of oxycodone was 50 mg (range: 40.0–62.4 mg) and fentanyl was 0.8 mg (range: 0.6–1.1 mg), with a potency ratio of 62.5:1.

**Figure 1:**
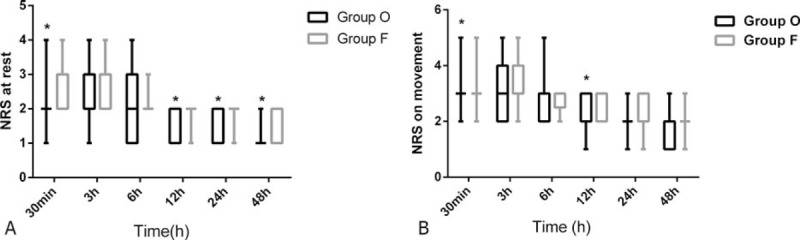
RR in the oxycodone group and intoe fentanyl group at 30 minutes, 3, 6, 12, 24, and 48 hours after the surgery. Results are shown as boxplots with mean (SD). There were no significant differences between the 2 groups. F = fentanyl, O = oxycodone, RR = respiration rate, SD = standard deviation.

NRS at rest was significantly lower in the group O at 30 min, 12, 24, and 48 hours after operation (*P* *<* 0.05, respectively) (Fig. 1A; left panel) and NRS upon movement was significantly lower in the group O at 30 minutes, 12 hours after the surgery (*P* = 0.04, 0.01, respectively) (Fig. 1B; right panel). Three patients in Group O and 5 patients in group F reported insufficient analgesia and requested additional analgesics, but the differences were not significant (*P* = 0.79).

Main adverse events are reported in (Table 2). The percentage of patients experienced at least 1 adverse event were higher in group O than in group F, but the differences were not significant (33.3% vs 27.6%, *P* = 0.64). Three subjects in group O (11.1%) and 9 subjects in group F (31.0%) had a sedation score of 4 after 30 minutes of surgery, and the sedation scores 30 minutes after the surgery was significantly higher in group F than in group O (*P* = 0.04). There were no statistically significant intergroup differences regarding the incidence and severity of dizziness, nausea and vomiting. In group O, however, 1 patient experienced moderate vomiting and was administered 2 doses of metoclopramide 10 mg i.m., and 1 patient experienced severe vomiting and requested terminating PCA use.

**Table 2:**
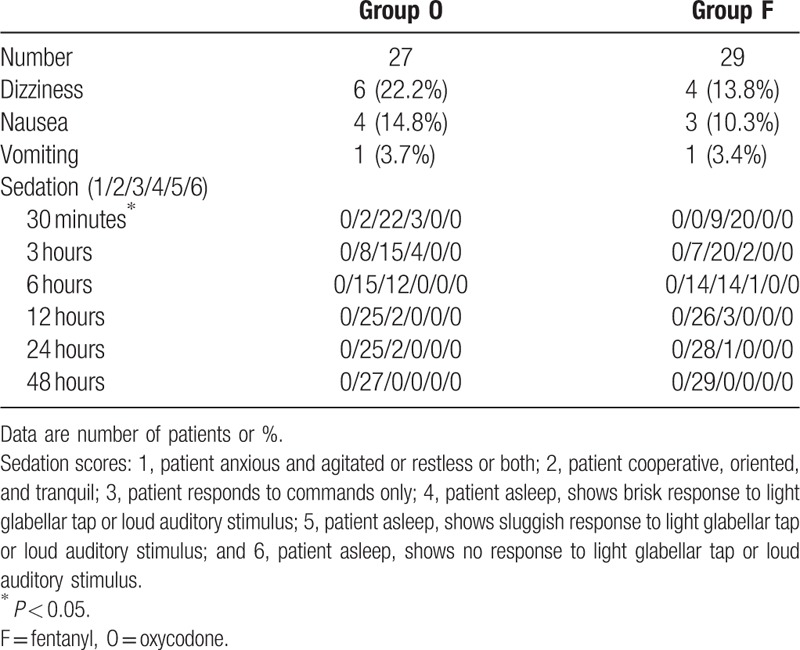
Incidence of postoperative adverse events during 48 hours.

**Figure 2:**
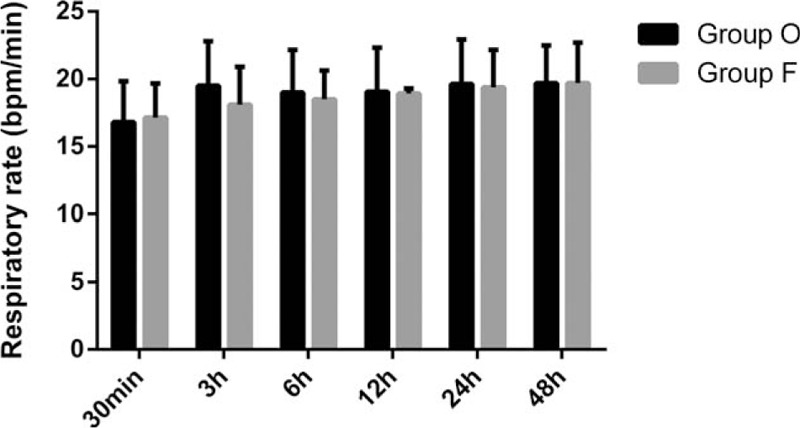
Resting NRS (A) and moving NRS (B) scores at 30 minutes, 3, 6, 12, 24, and 48 hours after the surgery. Results are shown as boxplots with medians represented by horizontal lines with the max at the top and the min at the bottom. ^∗^*P* < 0.05. F = fentanyl, NRS = numeric rating scale, O = oxycodone, RR = respiration rate, SD = standard deviation.

RR at each time point were presented in (Fig. 2). No statistically significant differences between patients administered oxycodone and fentanyl were observed with regard to RR and no one reported respiratory depression in both groups.

The overall satisfaction with pain management was rated by patients at 48 hours after the surgery, and there was no statistically significant difference between the 2 groups (*P* = 0.15) (Table 3).

**Table 3:**
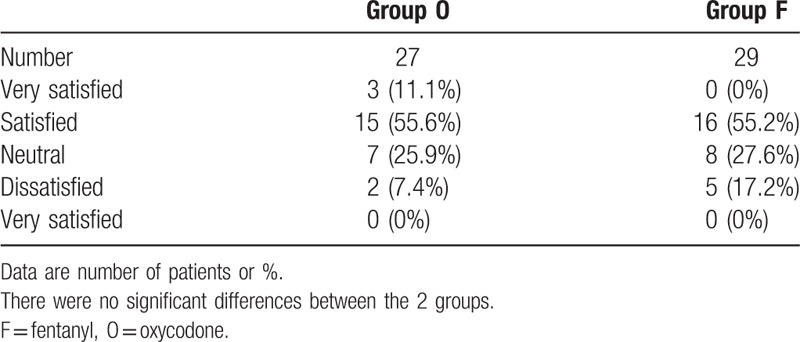
Patient satisfaction at 48 hours postoperatively.

## 4 Discussion

This study shows that oxycodone at a cumulative median dosage of 50 mg (range: 40.0–62.4 mg) provided better analgesia than fentanyl at a cumulative median dosage of 0.8 mg (range: 0.6–1.1 mg) over 48 hours after gastric laparotomy.

Oxycodone is a potent opioid, which may make it useful in the postsurgical pain control. The onset time of i.v. oxycodone is 2 to 3 minutes after injection and duration of action is 4 hours 52 minutes after a single dose of i.v. Oxycodone. Many studies have suggested that intravenous oxycodone is an effective treatment for acute postoperative pain.^[14–17]^ In this study, we planned to evaluate the analgesic efficacy and tolerability of intravenous oxycodone and fentanyl using PCA in patients after gastric surgery.

However, the safe dose conversion ratio of oxycodone to fentanyl is yet to be established. In the koch et al Study,^[18]^ 78 laparoscopic cholecystectomy patients were randomized to receive IV oxycodone or IV fentanyl for pain control at the end of anesthesia and in the postanesthetic care unit, the median intraoperative and postoperative opioid consumption was 15 mg oxycodone (range 10–40 mg) and 200 μg fentanyl (range 100–500 μg), respectively. The oxycodone patients experienced better analgesia but also more side effects. In a subsequent study in patients scheduled for laparoscopic cholecystectomy, IV-PCA oxycodone provided comparable effects for pain relief compared to IV-PCA fentanyl in a potency ratio of 1:75.^[19]^ In a study of 60 laparoscopic hysterectomy, patients were randomized to receive PCA oxycodone or PCA fentanyl (potency ratio 100:1) following surgery find that oxycodone presented a better analgesic effect, also resulted in a higher occurrence of postoperative nausea and vomiting.^[20]^ In another recent similar study, the potency ratio was 60:1, and both agents were found to provide similar analgesic efficacy and similar rates of adverse events.^[21]^ Overall, the potency ratio of fentanyl to morphine has a range of 60–100:1. After conducting a series of pilot study, we originally performed this study under the hypothesis that the potency ratio of oxycodone to fentanyl was 60:1.

It has been suggested that the most effective method to determine adequate doses in relation to the present pain is titrating the dose, and IV titrating has been used for rapid opioid titration in many clinical situations. Patient-controlled techniques allow patients to self-administer small boluses of analgesic, and thus providing better titration. Overall, in our study the potency ratio of fentanyl and oxycodone was 62.5:1, the results from the present study compare favorably with those from the fentanyl/oxycodone study after laparoscopic gynecological surgery for the potency ratio of 60:1, and was lower with all other previous studies. This seems to suggest that oxycodone is more potent than morphine. Our finding is in line with previous studies that have also suggested that the potency ratio of oxycodone to morphine is more on the order of 1:1.3–1.5.^[13,17]^ However, IV oxycodone and IV morphine were thought to be equipotent, that is, had a potency ratio of 1:1 in many studies.^[14,16]^ The pain mechanism might make the difference. Gastric laparotomy is often accompanied by severe visceral pain. Traditional μ-opioid agonists, such as fentanyl, however, may not be optimal in the treatment of visceral pain. Oxycodone is a semisynthetic derivative of thebaine. The main mechanism of action of oxycodone is associated with stimulation of the peripheral and central opioid receptors of the μ and κ type. It has been suggested that κ receptors constitute an essential part in the analgesic mechanism of action of oxycodone. Opioid receptors of the κ type on peripheral nerves may be an important feature for antinociception in the visceral pain system, and hence oxycodone has high therapeutic efficacy, especially in visceral pain,^[7]^ but this may not be the case for other types of pain. Therefore, Oxycodone may have more of an advantage over other opioids at equianalgesic dosages such as morphine after surgeries in which the visceral pain component is a large contributor to a patient's overall postoperative pain.^[13,22]^

Opioids are the most effective drugs for the management of moderate-to-severe perioperative pain in the major abdominal surgeries. However, their adverse effects have limited the use of them as analgesic agents. Therefore, ketorolac was added to the opioid IV-PCA in an effort to maintain the analgesic efficacy of opioids while reducing their adverse effects. Ketorolac have been shown to reduce postoperative opioid consumption while maintaining pain control and did not increase the incidence of non-steroidal anti-inflammatory drugs (NSAIDs)-related side effects.^[23,24]^ In addition, some reports have suggested that the administration of cyclooxygenase (COX)-2 inhibitors can be effective in reducing movement-evoked pain by preventing central sensitization.^[25]^ Furthermore, the addition of ketorolac in intravenous PCA opioid could shorten the recovery duration of postoperative bowel movement and passage of fatus in patients who received major colorectal surgeries.^[23,24]^

Our results showed that although the difference was statistically insignificant, the oxycodone group experienced more side effects, and one patient in group O alone requested additional antiemetics, this result was consistent with the report that oxycodone had a tendency toward more side effects than fentanyl.^[18,20,21]^

In this study, we also found that the sedation scores 30 minutes after the surgery was significantly higher in group F than in group O. There are possible explanations to this finding. First, it has been demonstrated that oxycodone produced similar or less sedation compared to fentanyl in several recent studies.^[18,20,21]^ In addition, median doses of opioid consumed 30 minutes postsurgery was oxycodone 3.5 mg (range: 2.4–5.4 mg) and fentanyl 64 μg (range: 49–120 μg), this meant to be relatively few oxycodone consumption during this time period.

There was no statistically significant difference between the 2 groups in terms of satisfaction with the IV-PCA 48 hours after the surgery. However, 2 subjects in group O against five subjects in group F reported disappointment with the pain control. These results may indicate that although the oxycodone group experienced more side effects, patient's satisfaction with the good analgesic effect presented by oxycodone make up this defect.

In conclusion, this study demonstrated that by PCA-based drug administration, oxycodone was comparable to fentanyl when used with a conversion ratio of 62.5:1 in patients following gastric laparotomy. However, clinical data indicate that not only the type of surgery but also the extent of surgery may affect the comparison of different opioid analgesics. Future studies in various clinical settings are warranted to investigate the efficacy and tolerability of IV oxycodone versus IV fentanyl, and the appropriate equianalgesic dose ratio of IV oxycodone and IV fentanyl.
